# Bacterial biota composition in gut regions of black soldier fly larvae reared on industrial residual streams: revealing community dynamics along its intestinal tract

**DOI:** 10.3389/fmicb.2023.1276187

**Published:** 2023-12-01

**Authors:** Dries Vandeweyer, Daniele Bruno, Marco Bonelli, Freek IJdema, Bart Lievens, Sam Crauwels, Morena Casartelli, Gianluca Tettamanti, Jeroen De Smet

**Affiliations:** ^1^Research Group for Insect Production and Processing, Department of Microbial and Molecular Systems, KU Leuven, Geel, Belgium; ^2^Department of Biotechnology and Life Sciences, University of Insubria, Varese, Italy; ^3^Department of Biosciences, University of Milan, Milan, Italy; ^4^Laboratory for Process Microbial Ecology and Bioinspirational Management, Department of Microbial and Molecular Systems, KU Leuven, Leuven, Belgium; ^5^Interuniversity Center for Studies on Bioinspired Agro-environmental Technology, University of Naples Federico II, Portici, Italy

**Keywords:** *Hermetia illucens*, gut regions, hindgut, bacterial community, microbiota, Illumina MiSeq

## Abstract

Some insect species have gained attention as efficient bioconverters of low-value organic substrates (i.e., residual streams) into high-value biomass. Black soldier fly (BSF) (*Hermetia illucens*) larvae are particularly interesting for bioconversion due to their ability to grow on a wide range of substrates, including low-value industrial residual streams. This is in part due to the plasticity of the gut microbiota of polyphagous insects, like BSF. Gut microbiota composition varies depending on rearing substrates, via a mechanism that might support the recruitment of microorganisms that facilitate digestion of a specific substrate. At the same time, specific microbial genera do persist on different substrates via unknown mechanisms. This study aimed to offer insights on this microbial plasticity by investigating how the composition of the bacterial community present in the gut of BSF larvae responds to two industrial residual streams: swill (a mixture of catering and supermarket leftovers) and distiller’s dried grains with solubles. The bacterial biota composition of substrates, whole larvae at the beginning of the rearing period and at harvest, rearing residues, and larval gut regions were investigated through 16S rRNA gene sequencing. It was observed that both substrate and insect development influenced the bacterial composition of the whole larvae. Zooming in on the gut regions, there was a clear shift in community composition from a higher to a lower diversity between the anterior/middle midgut and the posterior midgut/hindgut, indicating a selective pressure occurring in the middle midgut region. Additionally, the abundance of the bacterial biota was always high in the hindgut, while its diversity was relatively low. Even more, the bacterial community in the hindgut was found to be relatively more conserved over the different substrates, harboring members of the BSF core microbiota. We postulate a potential role of the hindgut as a reservoir for insect-associated microbes. This warrants further research on that underexplored region of the intestinal tract. Overall, these findings contribute to our understanding of the bacterial biota structure and dynamics along the intestinal tract, which can aid microbiome engineering efforts to enhance larval performance on (industrial) residual streams.

## Introduction

In recent years, the potential of insects as efficient bioconverters of low-value organic substrates into food, feed and biomolecules has come into focus (De Smet et al., 2018; Varelas, 2019). Within the concept of circular economy, insect-mediated bioconversion has gained specific attention, especially for the reuse and valorization of low-value residual streams from industry or generated by human consumption (Jiang et al., 2019). One of the most interesting insect species for such bioconversion is the black soldier fly (BSF, *Hermetia illucens*) (Tomberlin and van Huis, 2020).

Although BSF larvae are capable of growing on a large variety of substrates, including economically relevant low-value organic residual streams such as brewer’s spent grains or catering waste (Zheng et al., 2012; Ceccotti et al., 2022), their performance is highly substrate-dependent (Broeckx et al., 2021). For example, rearing parameters such as larval development time and maximal larval weight are strongly influenced by the rearing substrate used (Cappellozza et al., 2019). Moreover, the diet affects larval gut microbiota counts and dynamics (Bruno et al., 2019b; Wynants et al., 2019; Klammsteiner et al., 2020), raising the question if and how microbiota changes are correlated with changes in growth performance (Klammsteiner et al., 2021). In this regard, research into the metabolic mechanisms contributing to insect bioconversion abilities carried out by the insect itself and its gut microbial communities has been initiated (Jiang et al., 2019). Recently, the use of germfree and gnotobiotic BSF larvae has already illustrated how the gut microbiota aids in protein degradation in these larvae (Yu et al., 2023). An in-depth knowledge on the interplay between insect and microbes for metabolic processes is crucial for the development of microbiome engineering tools to boost larval performance on such industrial residual streams, in turn increasing the sustainability of BSF larvae rearing.

Most studies, including those mentioned above, have mapped how the gut microbiota changes based on the rearing substrate at the whole larvae or whole gut level (Wynants et al., 2019; Klammsteiner et al., 2020; Tegtmeier et al., 2021a,b; Zhineng et al., 2021; Gorrens et al., 2022). However, Bonelli et al. (2019) revealed that the gut of the BSF larvae is organized in distinct regions with distinct morphology and function. Briefly, the alimentary canal can be divided into a short foregut, a midgut consisting of three main regions (anterior, middle, and posterior), and a hindgut. A focused analysis on the three midgut regions revealed differences in enzyme activity, luminal pH, and epithelium morphology (Bonelli et al., 2019). Hence, the gut should be considered as a collection of micro-environments, each harboring specific microbiota. Indeed, it has been found that in BSF larvae the three midgut regions harbor different microbial communities related to, or influenced by, the chemical properties of the midgut lumen and the physiological functions of each region (Bruno et al., 2019b). Characterizing the microbiota within these distinct regions, compared to looking at the whole gut level, offers the opportunity to simplify the correlation of the presence of specific microorganisms in a certain region with their functions to support the host in the digestive processes active in that specific gut region. This approach, together with *in vitro* and *in vivo* work necessary to validate such correlations, will allow to define the contribution of the microbial community and that of the insect itself in the digestive processes, and to identify specific bacteria and strains endowed with functions for the degradation of specific polymers (e.g., cellulose, hemicellulose, and lignin). In addition, it will contribute to understand how the diet shapes the microbiota and if possible variations in the microbial community composition may be responsible for different larval growth performance (Tettamanti et al., 2022).

The objectives of this study were to generate additional data sets on the microbial community present (1) in whole BSF larvae during their rearing on two relevant industrial residual streams, i.e., a mixture of catering and supermarket food leftovers and distiller’s dried grains with solubles, and (2) in the gut regions of the BSF larvae at harvest, reared on the same residual streams. Using the same sequencing pipeline as employed in previous studies (Wynants et al., 2019; IJdema et al., 2022), the microbiota of whole larvae as well as that of gut regions was described, taking into account larval growth performance and the microbial and nutritional composition of the selected residual streams. Additionally, an analysis of the microbial composition in the hindgut was included, as the community in this region had not been analyzed to date. This study increases the existing body of information on the BSF larval microbiota and such data represent the first step to correlate the presence of specific microorganisms with metabolic functions in the gut.

## Materials and methods

### Insect rearing substrates, rearing protocol, and larval growth monitoring

To compare the impact of two different industrial residual streams on larval development and their gut microbiota, BSF larvae were reared on a catering and supermarket food leftover mixture (further referred to as “swill”) and on dried distiller’s grains with solubles (DDGS, brand Proticorn) a grain-based residual stream from biofuel production. Swill and DDGS were directly obtained from industrial companies, more precisely Renewi (Kampenhout, Belgium) and Alco Bio Fuel (Ghent, Belgium), respectively. Although swill was likely predominantly plant-based, its exact composition remains unknown, as it was sourced from various catering companies and supermarkets by Renewi. Commercial chicken feed (CF, Chicken start mash, AVEVE, Leuven, Belgium) was used as control substrate. Swill was stored frozen (−20°C) until use. Prior to rearing, it was thawed at room temperature, drained to remove excess free water, and then directly provided to the larvae, while CF and DDGS were mixed in a ratio 1:1 dry matter:water.

**FIGURE 1:**
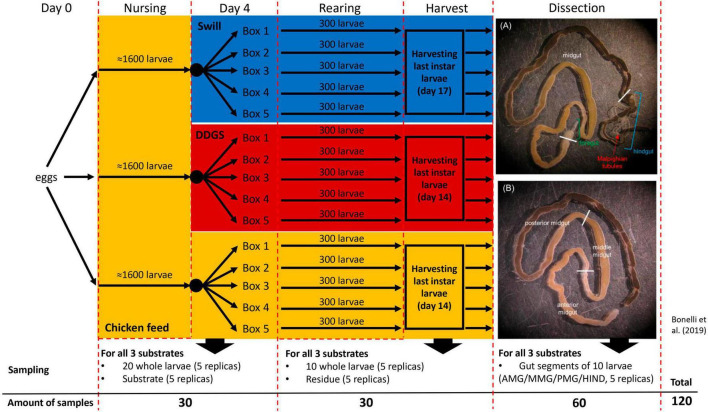
Experimental set-up of black soldier fly rearing on two industrial residual streams and chicken feed as control. The overview contains the sampling details at day 4 of rearing, at harvest and after dissection of the larvae to obtain the three main gut sections **(A)** and the middle midgut sections **(B)**.

Eggs, obtained from a BSF colony established in 2015 at University of Insubria (Varese, Italy) (Bruno et al., 2019a), were allowed to hatch at 27.0 ± 0.5°C, 70 ± 5% relative humidity, in the dark. For each rearing substrate, neonate larvae were nursed for 4 days on CF. At day 4, the larvae were divided into five separate rearing boxes (16 cm × 16 cm × 9 cm) (300 larvae each) (Figure 1) for each diet, filled with 100 g of substrate (CF, swill or DDGS, prepared as described above) and reared at 27.0 ± 0.5°C, 70 ± 5% relative humidity, in the dark, until reaching the last larval instar. Fresh diet was added to the feeding substrate every 3 days.

To monitor larval growth on the three substrates over time, random samples of at least 20 larvae were taken each 2 days starting at day 4. After sampling, the larvae were washed in tap water to remove food debris from the body, wiped dry, and then weighed. Weights were recorded until 25% of insects reached the pupal stage. The analysis of larval growth on each substrate was conducted in triplicate.

### Experimental set-up and sampling

A complete overview of the experimental set-up is given in Figure 1. During rearing, two sampling time points were selected to monitor the bacterial biota. At day 4, 5 samples, each consisting of 20 larvae, were collected from all three separate nursing boxes. Additionally, 5 samples of 5 g for each of the three different rearing substrates (before administration to the larvae) were taken. The second sampling time point (denoted as “larvae at harvest”) was at day 14 for larvae reared on CF and DDGS, and at day 17 for those reared on swill (corresponding to the last day of the larval cycle, when the larvae reached their maximum weight and were still actively feeding, before prepupal phase). At this time point, one sample consisting of 10 fully grown larvae, and one sample of 5 g of rearing residue (residual substrate at the end of the rearing period, including feces and exuviae) was collected from each box per rearing substrate. After sampling, larvae were washed with 70% ethanol in water and then 2 times in autoclaved distilled water to remove any excess of food from the cuticle. At that second sampling time point, one additional pool of 10 larvae was collected from each rearing box per substrate. These larvae were washed, disinfected (see above), and dissected to obtain the four different gut regions (see “*Dissection and isolation of gut regions*” below). As such, 20 samples of gut regions were obtained for each rearing substrate. In total 120 samples were collected to be analyzed for bacterial biota composition (Figure 1). Samples were stored frozen (−20°C) until analysis.

### Determination of substrate chemical composition

The chemical composition analysis of the rearing substrates was conducted at La-Chi laboratory (Department of Agronomy, Food, Natural Resources, Animals and Environment, University of Padua, Agripolis, Legnaro, Italy). Samples of CF and DDGS were analyzed as they were, while swill samples were lyophilized with a freeze-dryer (Alpha 2-4 LD plus, Martin Christ GmbH, Osterode am Harz, Germany) at 12–15 mbar and −80°C overnight. All samples were analyzed for crude protein, crude lipid, crude fiber, nitrogen-free extract, and ash following the protocols of AOAC International (Horwitz, 2000; Latimer, 2016).

### Dissection and isolation of gut regions

To obtain the four gut regions, larvae at harvest were first anesthetized on ice and then dissected to isolate the gut under a stereomicroscope in a horizontal-flow hood, by using sterile tweezers and scissors to avoid cross-contamination of the samples. The gut was placed in a sterile Petri dish filled with sterile phosphate-buffered saline (in mM: 137 NaCl, 2.7 KCl, 4.3 Na_2_HPO_4_, and 1.4 KH_2_PO_4_; pH 7.4) and then divided into the four regions of interest: the anterior, middle, and posterior midgut (AMG, MMG, and PMG, respectively) and the hindgut (HG) (Bonelli et al., 2019). To reduce potential external contaminations, a new Petri dish was used for the dissection of the gut from each larva, and tweezers and scissors were disinfected with 70% ethanol in water between each dissection. Pools of 10 gut regions were collected (for 5 replicates per gut regions per diet) in sterile plastic tubes and stored at −80°C until use.

### DNA extraction and total bacterial load qPCR assay

Prior to DNA extraction, all samples (120 in total) were thawed for 1 h in a refrigerator at 4°C. The samples were homogenized by either mixing them with a sterile spatula [fresh substrates (15 samples of 5 g) and rearing residues (15 samples of 5 g)] or crushing them in a sterile stomacher bag (fully grown larvae, 15 samples of 10 larvae each) or in a sterile centrifuge tube [neonates (15 samples of 20 larvae each) and gut regions (60 samples of 10 gut regions each)]. Samples were subjected to genomic DNA extraction using the E.Z.N.A.^®^ Soil DNA kit (Omega Bio-Tek, VWR, Leuven, Belgium), according to the manufacturer’s protocol. DNA quantity and quality was assessed using a mySPEC spectrophotometer (mySPEC Touch, 732-2534, VWR) prior to storing them at 20°C until further analysis.

To quantify the total bacterial load of the samples, DNA extracts were subjected to quantitative real-time PCR (qPCR), targeting the V4 region of the 16S rRNA gene, as described by Borremans et al. (2019). Each DNA extract was measured twice. Briefly, qPCR reactions (10 μl) contained 1 μl DNA extract (with DNA concentration of 8 to 413 ng/μl, on average 67 ng/μl), 5 μl PowerUp™ SYBR^®^ Green qPCR master mix (Life Technologies, Thermo Fisher Scientific, Asse, Belgium) and 0.2 μM of each primer (515F and 806R) (Supplementary Table 1). qPCR was executed using a QuantStudio 3 system (Applied Biosciences, Thermo Fisher Scientific). The reaction protocol consisted of one cycle of 2 min at 50°C and 10 min at 95°C, followed by 40 cycles of 15 s at 95°C and 60 s at 60°C. Fluorescence was measured at the end of each cycle and the assay was followed by a melting curve analysis while gradually increasing temperature from 60 to 95°C (0.1°C/s). Each assay contained a no template control (NTC, sterile milli-Q water instead of DNA extract) and a serial standard dilution of purified (E.Z.N.A.^®^ Cycle Pure kit, VWR) and quantified (mySPEC Touch, VWR and DNA Copy Number Calculator, Thermo Fisher Scientific) PCR products of the 16S rRNA gene fragment (515F and 806R) generated from a sample of whole larvae reared on chicken feed (WLD18CF1) (Supplementary Table 1). The gene copy number for each sample was calculated using the Design and Analysis 2 application in the DataConnect™ Cloud Software (Thermo Fisher Scientific) and expressed as gene copies/g sample (mean of both replicates), thus correcting for unequal sampling mass. Depending on the qPCR run, a Cq cut-off of 30, 31 or 34.5 was employed for positivity, corresponding with a detection limit of 100 or 1,000 copies/μl DNA extract. Since certain bacterial species contain multiple copies of the 16S rRNA gene, which may lead to overestimation of absolute abundances, normalization of the qPCR results is sometimes applied. However, this practice is still questioned (Starke et al., 2021), and therefore not implemented in this study.

### Determination of bacterial biota composition via 16S rRNA gene sequencing

To obtain an overview of the bacterial biota composition, each sample was subjected to Illumina MiSeq sequencing with dual indexing strategy (Kozich et al., 2013), as described by Bossaert et al. (2021). As preparation, the V4 16S rRNA gene region of all DNA extracts was amplified by PCR using barcoded versions of the primers 515F and 806R (Supplementary Table 1) (Caporaso et al., 2011; Kozich et al., 2013). Next, obtained amplicons were purified using Agencourt AMPure XP magnetic beads (Beckman Coulter, Indianapolis, IN, USA; manufacturer’s protocol) and pooled into a library at equimolar concentrations. Negative controls from the PCR runs, as well as a mock community (Gloder et al., 2021) composed of seven bacterial species isolated from insects (Supplementary Table 2), were included in the library. After final purification by ethanol precipitation and gel extraction (QIAquick Gel Extraction Kit, Qiagen, Hilden, Germany), the library was diluted to 2 nM and sequenced at the University of Antwerp, using an Illumina MiSeq sequencer with a MiSeq v2 500-cycle reagent kit (Illumina, San Diego, CA, USA).

In the resulting demultiplexed FASTQ sequencing file, barcodes and primer sequences were removed. To form consensus sequences, paired-end reads were merged using USEARCH (v11.0.667) (Edgar, 2013) with a maximum of 10 mismatches allowed in the overlap region. Next, sequences were truncated at the 250^th^ base and reads shorter than 250 bp or reads with a total expected error threshold above 0.1 were discarded using USEARCH. Subsequently, Mothur’s (v1.39.3) commands “classify.seqs” and “remove.lineage” or “get.lineage” in combination with the SILVA database (v1.38) were used to identify and remove potential chloroplast, mitochondrial or other non-target sequences. Next, sequences were classified into zero-radius operational taxonomic units (zOTUs) (Edgar, 2016) by the UNOISE3 algorithm as implemented in USEARCH (Edgar and Flyvbjerg, 2015). Only zOTUs with a minimum global relative abundance of eight reads were kept and chimeric sequences were removed. All data sets were analyzed in R (v3.5.2) using “decontam” (v1.2.0) to correct for the presence of contaminants based on zOTU prevalence in insect samples versus the negative control samples (R Development Core Team, 2013; Davis et al., 2018). Finally, the number of sequences was rarefied to 13,000 reads, while excluding relative abundances below 0.1%. The taxonomic origin of each zOTU was determined with the SINTAX algorithm as implemented in USEARCH based on the SILVA Living Tree Project v1.23 (LTP v1.23) (Supplementary Table 3). Taxonomic assignments were considered reliable when bootstrap confidence values exceeded 0.80. Additionally, taxonomic identification of the zOTUs remaining after decontamination was verified with a BLAST search against type materials in GenBank (Supplementary Table 3). Analysis of the mock community demonstrated that all expected taxa were found, and no single contaminant had passed the quality filtering and decontamination steps, indicating that the experimental conditions were met to achieve robust data. From the processed sequencing data, Chao1 and Shannon-Wiener diversity indices were calculated using the Phyloseq (v1.26.0) package in R (McMurdie and Holmes, 2013).

### Statistical analyses and data visualization

To display total bacterial load and diversity indices of the BSF larvae gut regions, boxplot figures were designed in JMP Pro 17 (SAS Institute, v17.0.0). Differences between samples per rearing substrate in terms of mean total bacterial load and diversity were determined with non-parametric Kruskal–Wallis tests. Heatmaps of the most abundant zOTUs were generated using the plot_heatmap function in the phyloseq package in R. Where applicable, pairwise comparison was performed using Dunn’s *post-hoc* tests. In all cases, a significance level of 0.05 was considered (JMP Pro 16). To visualize differences in bacterial biota composition between samples, non-metric multidimensional scaling (NMDS) ordination plots, based on Bray–Curtis distances, were generated by using the plot_ordination function within the phyloseq package in R. Bray–Curtis dissimilarities of Hellinger-transformed relative abundance data were used to statistically test the group-level effects in the microbial communities, using PERMANOVA (9999 permutations) with the adonis2 function in the vegan package in R (Oksanen et al., 2022) (Supplementary Table 5). Discriminant analysis effect size (LEfSe), DESEq2 and EdgeR BioMarker analyses were conducted to search for distinctive zOTUs in the larval gut per diet using the run_lefse, rune_deseq2 and run_edger commands with default settings in the MicrobiomeMarker packge in R. Additionally, a heatmap of Z-scores for the most abundant zOTUs, including dendrogram, was plotted using the gplots package in R (Warnes et al., 2016). Finally, to detect differences in terms of final larval weight and length of the larval period between insects reared on the three substrates, non-parametric Kruskal–Wallis followed by Dunn’s *post-hoc* tests and one-way analysis of variance (ANOVA) followed by Tukey’s test were performed in R environment, respectively.

## Results and discussion

### Nutritional composition of rearing substrates

To allow the assessment of how changes in the microbiota might be correlated to specific characteristics of the diets, their nutritional composition was determined. This analysis revealed a strong variation among chicken feed and the two industrial residual streams that served as rearing substrates in this study (Table 1). The diet with the highest content in protein on “as fed” basis was DDGS, followed by CF and swill (Table 1). The total amount of lipids on “as fed” basis in DDGS (4.5%) was almost double compared to swill and CF (2.5 and 2.6%, respectively). The crude fiber content was low in all three diets compared to other tested substrates in literature (Broeckx et al., 2021). As fed, DDGS contained 1.8% crude fiber, which was slightly higher compared to the swill (0.9%) and CF (1.0%) substrates. Finally, the following order was observed in the nitrogen-free extracts (which included, among other chemicals, starch and sugars) of the three diets: CF > DDGS > swill (respectively, 26.6, 18.0, and 7.4%).

**TABLE 1:** Chemical composition and moisture content of chicken feed, DDGS, and swill. Values (g per 100 g of diet) are expressed on “as fed” and “dry matter” bases. The former is computed on the diet as fed to the larvae taking into consideration the water content, the latter refers to a moisture-free basis.

Component	Chicken feed		DDGS		Swill	
	As fed	Dry matter	As fed	Dry matter	As fed	Dry matter
Crude protein	8.1	20.0	11.1	29.8	2.2	16.2
Crude lipids	2.6	6.3	4.5	12.1	2.5	17.8
Crude fiber^a^	1.0	2.6	1.8	4.9	0.9	6.5
Nitrogen-free extract^b^	26.6	65.4	18.0	48.4	7.4	53.4
Ash	2.3	5.7	1.8	4.8	0.8	6.1
Water content	59.4	–	62.8	–	86.2	–

^*a*^Includes most of cellulose and insoluble lignin.

^*b*^Includes sugars, organic acids, pectins, soluble lignin, hemicellulose and a small percentage of cellulose.

Such differences in nutritional composition among diets can impact the growth performance of the larvae (Scala et al., 2020; Broeckx et al., 2021; Laganaro et al., 2021). For example, diets with a higher protein content led to heavier larvae with often reduced development time (Nguyen et al., 2013; Beniers and Graham, 2019; Eggink et al., 2023). At the same time, the ratio between proteins and carbohydrates has also been found to be essential for good larval growth, with an optimal range indicated between 1:2 and 1:3 (Eggink et al., 2023).

### Bacterial community composition of the rearing substrates

Aside from differences in nutritional composition, the diet (Scala et al., 2020; Broeckx et al., 2021; Laganaro et al., 2021) can also contain a variable microbial community. Together with the nutritional variation, this can affect the composition of the gut microbiota in the larvae. In particular, taxa with functions able to degrade specific macromolecules present in the rearing substrates may establish themselves in the larval gut community (Auger et al., 2023) and positively affect larval growth.

#### Bacterial load in the substrates over time

To get a first view on the microbial differences of the rearing substrates, the total bacterial load was determined. The initial bacterial load was much lower for DDGS than for CF and swill, being on average 3.3 × 10^5^ versus 3.2 × 10^8^ and 3.5 × 10^8^ 16S DNA copies/g substrate, respectively. As DDGS is the side-product of a fermentation process and underwent a drying step, such a reduction of microbial load was to be expected. Nevertheless it remains a good source of crude protein, fat, fiber, and small amounts of other nutrients such as vitamins (Iram et al., 2020). Hence, once rehydrated, microbes should proliferate well in the diet. Indeed, by the end of the experiment the total bacterial load in DDGS residue increased significantly to 2.1 × 10^9^ 16S DNA copies/g residue, which was similar to the mean DNA copy number/g in CF and swill residue (1.0 × 10^10^ and 3.1 × 10^8^, respectively). Interestingly, the total bacterial load in swill remained relatively constant over the experiment. A possible explanation might be the difference in moisture content (and thus water activity), which was already high for swill prior to sampling and use in our experiments. DDGS and CF, on the other hand, were dry formulas which required additional water at the onset of the experiment, likely generating more favorable conditions for microbial growth.

#### Microbial community composition in the substrates and its dynamics during BSF larvae rearing

**FIGURE 2:**
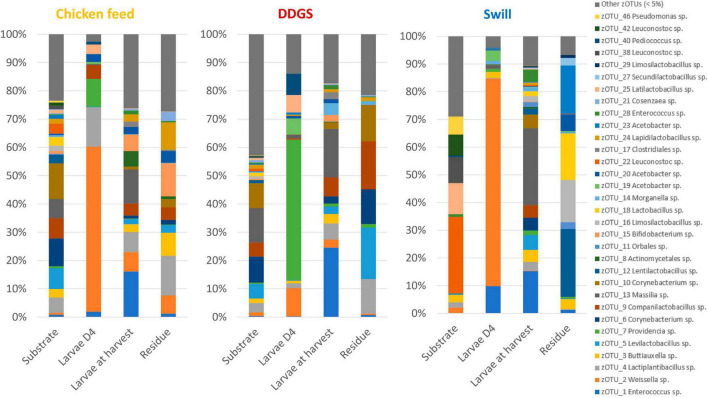
Relative abundance of the bacterial community composition of the substrate, the black soldier fly larvae at day 4 (D4) during rearing, the larvae at harvest and the residue after rearing, each for the three different substrates used in this study. Data are presented as mean of five replicas (*n* = 5), except for the chicken feed substrate (*n* = 1), the DDGS substrate (*n* = 4) and the larvae at harvest on DDGS (*n* = 3). Displayed zOTUs had an overall relative abundance above 5% in at least one sample. All other zOTUs are displayed as “Other zOTUs (< 5%)”. Identities of the zOTUs were obtained from the SILVA Living Tree Project.

Next, the diversity on a community level was explored using 16S rRNA gene sequencing. This revealed a more diverse microbiota in DDGS and CF diet in comparison to swill (Figure 2). This was shown by the lower mean Shannon diversity index for swill (3.18), compared to the ones for DDGS and CF (3.96 and 3.88, respectively) (Supplementary Table 4). This diversity declined in the residue for all three substrates to 2.89, 2.47, and 2.14 for CF, DDGS and swill, respectively. Jiang et al. (2019) observed a similar decline and they assumed this was due to the selective pressure exerted on the microbial community by the passage of substrate through the gut during feeding.

Four zOTUs were among the most abundant in both CF and DDGS (5–12% of all reads per zOTU). They were identified as a *Levilactobacillus* sp. (zOTU 5), two *Corynebacterium* sp. (zOTU 6 and 10), and a *Massilia* sp. (zOTU 13). On the other hand, the community in swill was dominated by lactic acid bacteria from the genera *Leuconostoc* (zOTUs 22, 38, 42, 56 and 58), which together accounted for 52.6% of all reads, and *Latilactobacillus* (zOTU 25, 11.2% of all reads). Based on this evidence, we can conclude that, from a microbiological perspective, DDGS and CF were more alike than swill. This may be explained by the fact that both DDGS and CF are grain-based substrates, rather than a mixture of food leftovers with a more variable composition. In the residue, shifts in this community occurred with zOTUs related to the larvae becoming more dominant over time. This is in line with previous studies on the impact of BSF larvae in shaping the microbial community of their residue (Jiang et al., 2019).

Next, we explored how these nutritional and microbial differences among the substrates impact the growth performance of the BSF larvae on these diets, and later on also their microbial community.

### Impact of the different rearing substrates on larval growth

**FIGURE 3:**
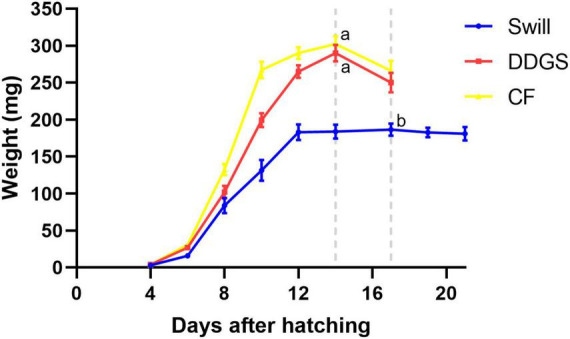
Development time of black soldier fly larvae reared on CF (yellow graph), DDGS (red graph), and swill (blue graph). Dotted gray lines refer to the day in which the larvae reached the maximum weight (considered the end of the larval stage). Error bars refer to standard error. Different letters indicate statistically significant differences between maximum weight reached by the larvae at the end of the larval stage, reared on the different substrates.

Important differences in larval growth performance were encountered among the rearing substrates used (Figure 3). In detail, larvae reared on DDGS, a grain-based by-product from biofuel production, showed a similar growth performance compared to the chicken feed substrate used as control, both in terms of length of the larval stage (14.3 ± 0.3 and 13.7 ± 0.3 days, respectively) (ANOVA *p*-value: *p* < 0.001, Tukey *p*-value: DDGS vs. CF, *p* = 0.392), as well as the maximum weight reached by the larvae (290 ± 11 mg and 303 ± 11 mg, respectively) (Kruskal–Wallis *p*-value: *p* < 0.001, Dunn *p*-value: DDGS vs. CF, *p* = 1.000). When larvae were reared on swill, a residual organic stream consisting of leftover food products collected from catering and supermarket, they grew slower (17.3 ± 0.3 days to complete the larval period) (ANOVA *p*-value: *p* < 0.001, Tukey *p*-value: swill vs. CF, *p* < 0.01) and remained smaller compared to the control (only 187 ± 8 mg as maximum weight reached by the larvae) (Kruskal–Wallis *p*-value: *p* < 0.001, Dunn *p*-value: swill vs. CF, *p* < 0.001). The comparison between these data on larval development and existing literature shows that larvae performed as expected on the swill diet. For example, Lievens et al. (2022) observed a maximum weight of 199.6 ± 16 mg when rearing BSF larvae on an artificial swill, composed from fresh ingredients rather than collected in industry. Another study that also examined an industrial food waste stream, reported a final mass of 176.4 ± 15 mg (Broeckx et al., 2021).

The negative effect on larval growth performance observed on swill may be explained by the lower amount of energy-containing macronutrients (proteins, fats, and carbohydrates) in this substrate, compared to the other diets (Table 1). Another possible parameter driving this difference in growth might be the high water content of swill (Table 1), since a high substrate moisture content, and more specifically an excess of free water, is detrimental for BSF larval growth (Padmanabha et al., 2020; Yakti et al., 2023). Keeping this in mind, it may still be interesting to use swill, potentially combined with fibers that have a high water binding capacity, as a rearing substrate due to its high availability, low cost, and the high upcycling value when it is used as insect rearing substrate compared to its current use in biogas production.

### Impact of rearing substrate and larval developmental stage on the bacterial biota composition of the whole larvae

In addition to its impact on the larval growth performance, the rearing substrate can affect the composition of the gut bacterial community of the larvae (Jeon et al., 2011; Bruno et al., 2019b). Specific gut microbes are involved in supporting the host in digestion processes thanks to the presence of genes encoding enzymes that allow the digestion of proteins, cellulose, lipids, pectin, or other macromolecules present in the diet (Engel and Moran, 2013; Callegari et al., 2020). Hence, the chemical content of the rearing substrates may impact on the composition of the microbial population in the larval gut (Bruno et al., 2019b; Klammsteiner et al., 2020) which, in turn, may affect the capability of the larvae to grow and bioconvert the substrate. Therefore, we first explored the microbiota of whole larvae during rearing on the selected substrates.

**FIGURE 4:**
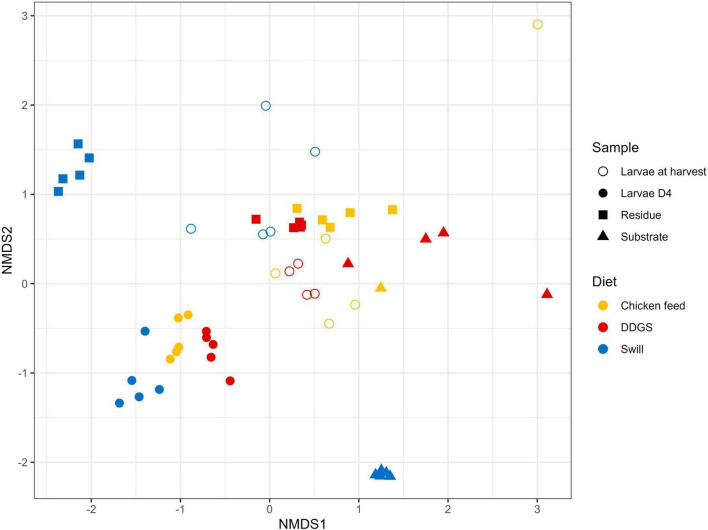
Non-metric multidimensional scaling (NMDS) plot based on Bray–Curtis dissimilarities derived from the Hellinger transformed relative abundance data of the bacterial communities (stress = 0.1757) of the substrate, the black soldier fly larvae at day 4 (D4) during rearing, the larvae at harvest and the residue after rearing, each for the three different rearing substrates used in this study (*n* = 5), except for the chicken feed substrate (*n* = 1), the DDGS substrate (*n* = 4) and the larvae at harvest on DDGS (*n* = 3). The greater the distance between two data points, the more dissimilar the bacterial communities. Different colors represent the different rearing substrates (yellow for chicken feed, red for DDGS, blue for swill), while different symbols represent the different sample types (triangle for substrate samples, balls for larvae samples at D4, circles for larvae samples at harvest and squares for residue samples).

The NMDS and PERMANOVA analysis on samples from substrates, rearing residues, and whole larvae at D4 and at harvest (Figure 4) showed a significant (*R*^2^ = 0.08; *p* < 0.001) impact of the diet on the bacterial composition of the larvae as well as variations between the different sample types (*R*^2^ = 0.15; *p* < 0.001). As expected, larval samples at D4 were closely related since they originated from larvae reared on the same diet (CF) and at the same day of development. On the contrary, the distance among samples at harvest, which originates from larvae reared on the three different diets since D4, increased. Surprisingly, the community of the larvae at D4 transferred to the DDGS differed relative to the communities at D4 of the larvae transferred to the other two diets (Figure 2). Most of the zOTUs identified were present in all three samples, which explains their close distance in the NMDS (Figure 4), yet the abundance of these zOTUs differed significantly between the three samples (Figure 2). For example, the ratio *Providencia* sp. (zOTU 7):*Weissella* sp. (zOTU 2) abundance for the larvae transferred to DDGS was about 5:1, while on the other two diets there was a ratio between 1:6 and 1:70 (Figure 2). As each diet was analyzed in an independent rearing cycle, this indicates that unknown environmental parameters may, even within biological replicates, alter the community dynamics and determine which member of the bacterial community becomes dominant. A similar variation in abundance was observed in the study of Gorrens et al. (2022), which looked at six consecutive rearing cycles in an industrial setting. At harvest, a shift was observed on all three substrates, thus also for the larvae grown on chicken feed (Figure 4). This is worth mentioning as these larvae remain on the same substrate their entire rearing cycle. This indicates that factors, like developmental stage, also have a clear impact on the composition of the bacterial community, aside from diet, as discussed below.

Zooming in on specific diet-dependent changes, it is interesting that zOTU 14, a *Morganella* sp., took up a considerable portion of the microbiota (4.1%) in the larvae at harvest especially on DDGS diet. *Morganella morganii*, together with *Klebsiella oxytoca*, has been linked in literature to aid the larvae of the tephritid fruit fly *Bactrocera dorsalis* in nitrogen recycling, by hydrolyzing urea in their gut (Ren et al., 2022). For this herbivorous insect, nitrogen is often the limiting nutrient, even more so due to the low nitrogen utilization in insects because of the excretion of large quantities of nitrogenous waste products. This excretion is mainly in the form of uric acid, although dipteran species degrade this uric acid into allantoin prior to excretion (Barragán-Fonseca et al., 2020). In nature, insects have therefore evolved a strategy using symbionts to recycle this nitrogenous waste (Douglas, 2015). Briefly, the uric acid or allantoin are metabolized into urea and then NH_4_^+^ by core symbionts, which also produce essential amino acids that can be reabsorbed by the insect (Green and Popa, 2012). While DDGS diet of course does not qualify as a nitrogen-limited diet, an unbalanced protein uptake is also shown to result in increased nitrogenous waste secretion in other insects, like the silkworm, *Bombyx mori* (Horie and Watanabe, 1983; Woods et al., 2019). Therefore, we hypothesize that the higher abundance of this *Morganella* sp. on this diet can be linked to the higher availability of a suited nitrogenous substrate to proliferate. Indeed, existing datasets also exhibit a higher abundance of this genus on protein rich diets, e.g., on fish diet (Bruno et al., 2019b). Furthermore, *Morganella* isolates from BSF larvae have already been shown to have urease activity (Callegari et al., 2020). Future functional characterization of these symbionts will hopefully be able to demonstrate their role in N waste metabolization.

Interestingly, a direct interaction between the microbiota of the substrate and the larvae was less apparent. While on CF several zOTUs [e.g., an *Enterococcus* sp. (zOTU 1), a *Massilia* sp. (zOTU 13) and a *Bifidobacterium* sp. (zOTU 15)] present in the substrate became more dominant in the larvae between D4 and harvest (Figure 2), other highly abundant bacteria in the different substrates were not necessarily found in high abundance after rearing the larvae on those substrates. For example, two main *Leuconostoc* species of the swill bacterial composition (zOTUs 38 and 42) were nearly absent in the larvae reared on this substrate. Consequently, the interaction between rearing substrate, microbiota, and larvae may depend on the specific microbial species, as it was observed for the fate of specific foodborne pathogens after inoculation in the substrate (De Smet et al., 2021; Gorrens et al., 2021).

In addition to the feeding substrate, another parameter shaping the microbial community in the larvae is insect development. In fact, a specific bacterial community composition has been shown to characterize different developmental stages in BSF (larvae, prepupae, pupae, adults, and eggs) (Zheng et al., 2013; Heussler et al., 2022) or even different larval instars (Gorrens et al., 2022; IJdema et al., 2022). As reported in Figures 2, 4 and as confirmed by the diversity indices (Supplementary Table 4) and an additional NMDS focused solely on the whole larvae samples (Supplementary Figure 1), the general microbial composition of larvae at day 4 (D4) and larvae at harvest showed large differences. However, it is clear that untangling the importance of both parameters, diet and insect development, on those differences is nearly impossible. This is in part due to the experimental set-up (Figure 1), where only the larvae reared on CF remain on the same diet for the whole experiment. Zooming in on that diet, larvae at D4 mainly harbored bacteria of the genus *Weissella* (zOTU 2) and *Providencia* (zOTU 7), while larvae at harvest contained a completely different and more diverse bacterial composition with other dominant genera like *Enterococcus* (zOTU 1), *Massilia* (zOTU 13), and *Levilactobacillus* (zOTU 4). This observation fits the hypothesis of a “maturation” of the bacterial community during the larval development. A similar maturation of bacterial community composition was not only observed in another study for BSF larvae (Gorrens et al., 2022), but is also known to occur in other livestock (Everaert et al., 2017) and humans (Beller et al., 2021).

An additional potential explanation for the alterations in the microbiota that occur from larvae D4 to larvae at harvest, independent of the type of diet, could be the modification of the rearing environment over time. In fact, the microbial composition of the substrate in which the larvae reside is strongly influenced by the presence of the larvae. Changes in substrate pH [typical evolution towards an alkaline pH (Meneguz et al., 2018)], presence of excrements, and transit through the larval digestive system may promote or avoid the growth of specific bacteria derived from the substrate (Bonelli et al., 2019; Bruno et al., 2019b). Indeed, a shift was observed in the bacterial composition from substrate to residue for all diets investigated in this study (*R*^2^ = 0.15, *p* < 0.001), as displayed in Figure 4 and Supplementary Figure 2. Also from Figure 2 and the diversity indices (Supplementary Table 4) it is clear that the residue from all rearing diets harbored a different and less diverse bacterial composition compared to the initial substrate.

### Bacterial community dynamics throughout the BSF larval gut regions

So far, the gut microbial community was evaluated at the level of the whole larvae, which offers the possibility to also look at the early stages of insect development when gut dissection is still too complex due to insect size. However, the diversity of the microbiota can be directly proportional to the complexity of the insect gut. In particular, variations in epithelium morphology, the presence of different pH in the lumen and/or digestive enzymes, aside from the type of ingested feed, can affect the composition of the microbiota along the whole insect gut (Dillon and Dillon, 2004). For BSF, the strong variation of pH in the larval gut (Bruno et al., 2019b), as well as the differences in the presence and activity of digestive enzymes (mainly lysozymes) (Bonelli et al., 2019, 2020) could strongly shape the microbial community along the whole length of this organ. Bruno et al. (2019b) were the first to explore this hypothesis. They found a decreasing diversity along the midgut regions, while bacterial load increased from anterior to posterior midgut. They also concluded that substrates with a high protein content could trigger midgut dysbiosis. To elaborate on these findings, this work also looked at additional substrates to test that assumption and included, for the first time, the analysis of the microbial community in the hindgut. In fact, to the best of our knowledge, the microbial composition of BSF larval gut was analyzed either on the whole organ (Klammsteiner et al., 2020; Shumo et al., 2021; Zhang et al., 2021; IJdema et al., 2022) or by subdividing the midgut in its three main regions (Bruno et al., 2019b). The introduction of the hindgut as separate sample provides new, interesting insights.

**FIGURE 5:**
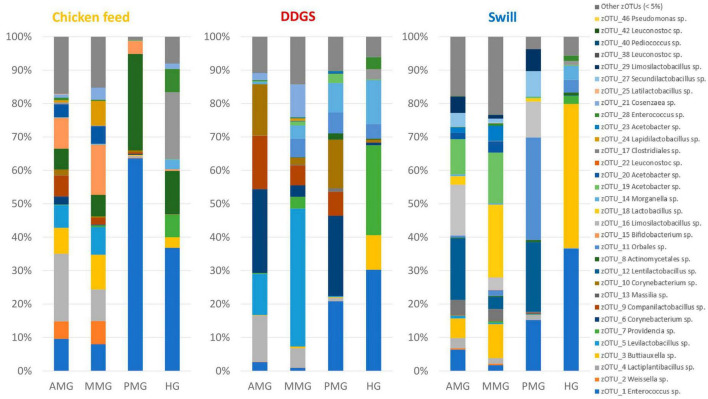
Relative abundance of the bacterial community composition of the black soldier fly gut regions, each for the three different rearing substrates used in this study. Data are presented as mean of five replicas. Displayed zOTUs had an overall relative abundance above 5% in at least one sample. All other zOTUs are displayed as “Other zOTUs (< 5%).” Identities of the zOTUs were obtained from the SILVA Living Tree Project.

**FIGURE 6:**
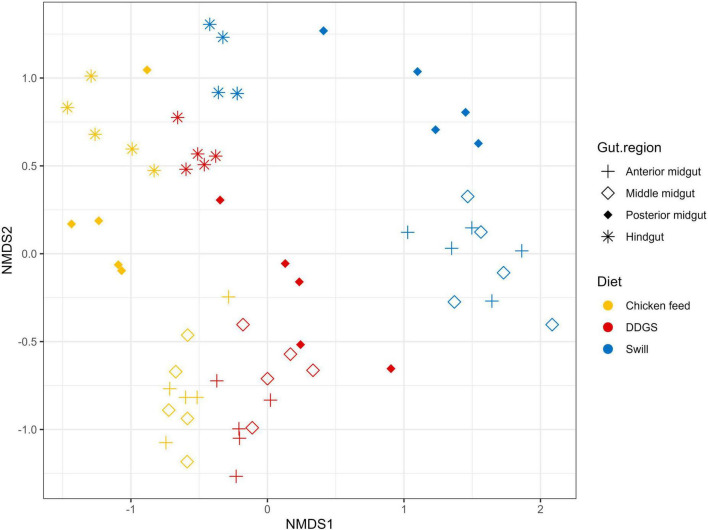
Non-metric multidimensional scaling (NMDS) plot based on Bray–Curtis dissimilarities derived from the Hellinger transformed relative abundance data of the bacterial communities (stress = 0.1677) of the black soldier fly gut regions, each for the three different substrates used in this study (*n* = 5). The greater the distance between two data points, the more dissimilar the bacterial communities. Different colors represent the different rearing substrates (yellow for chicken feed, red for DDGS, blue for swill), while different symbols represent the different sample types (plus for anterior midgut, open diamond for middle midgut, filled diamond for posterior midgut and asterisk for hindgut).

A comparison between the bacterial biota composition in the gut regions derived from BSF larvae reared on the three substrates used in this study revealed that a shift in bacterial biota occurred along the gut regions (*R*^2^ = 0.25; *p* < 0.0001) and across different diets (*R*^2^ = 0.26; *p* < 0.001). As displayed in Figures 5, 6, the composition of the AMG and MMG was, per substrate, still very similar. However, when comparing the PMG with the AMG and the MMG, a shift in bacterial composition was observed, as demonstrated by an increasing distance on the NMDS plot (Figure 6). In the PMG, typically a few bacteria became dominant. First, an *Enterococcus* sp. (zOTU 1) was found abundantly present in all PMG samples (64, 21, and 15% for PMGs from CF, DDGS, and swill, respectively), while this species was only detected in amounts lower than 10% in AMG and MMG samples. Other dominant zOTUs in PMG samples were an Actinomycetales sp. (zOTU 8, 29%) on CF as control, two *Corynebacterium* sp. (zOTU 6, 24% and zOTU 10, 15%) on DDGS, and a *Lentilactobacillus* sp. (zOTU 12, 21%), a *Limosilactobacillus* sp. (zOTU 16, 11%) and an Orbales sp. (zOTU 11, 31%) on swill. On the chicken feed and swill substrates, the main shift in bacterial composition occurred between the MMG and the PMG (Figure 6). This shift was also observed by Bruno et al. (2019b) and they hypothesized that the low pH (≤ 3) and the presence of antimicrobial peptides, lysozymes, and digestive enzymes in the MMG strongly affect a large fraction of living gut bacteria. Future research will have to explore if indeed this inactivation of microbes can be linked to these activities in the MMG. It is worth noting that our observations in the gut of larvae fed DDGS did not completely align with these observations, as the shift rather occurred only between PMG and HG (Figure 6). This seems to be correlated with the persisting presence of two *Corynebacterium* sp. (zOTU 6 and zOTU 10) and a *Companilactobacillus* sp. (zOTU 9) into the PMG.

Overall, the MMG selection seems to have least affected the lactic acid bacteria *Enterococcus* sp., *Lentilactobacillus* sp. and *Limosilactobacillus* sp., an Actinomycetales sp. and two *Corynebacterium* sp. from the Actinomycetia class, and an Orbales sp. in this study. Indeed, several lactic acid bacteria, including *Enterococcus* and (previously) *Lactobacillus* species, have been observed to survive very low pH levels (Menconi et al., 2014; Mubarak and Soraya, 2018) and also within the order of Actinomycetales, several acidophilic species can be identified (Guo et al., 2015). The order of Orbales is a relatively young bacterial order of Gammaproteobacteria. The first species in this order were identified in bumblebees and butterflies (Kwong and Moran, 2013) but, more recently, Orbales spp. were also associated with other insect species like darkling beetles and wild-caught *Drosophila* spp. (Martinson et al., 2017). While the survival at low pH was not yet clearly described for many Orbales sp., members of this order are responsible for the fermentation of polysaccharides in the bee gut. For example, a *Gilliamella* sp. is the primary degrader of pectin in the bee gut (Zheng et al., 2019). As this fermentation also triggers acid production, it is plausible to assume that these microbes have some resistance to withstand lower pH environments.

**FIGURE 7:**
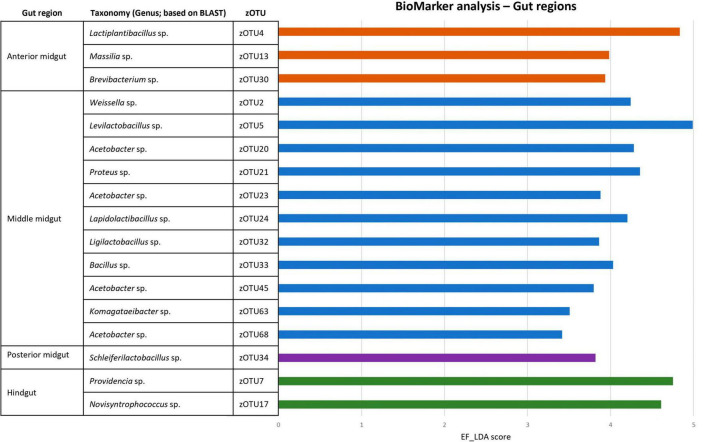
BioMarker analysis of different gut regions. Discriminant analysis effect size (LEfSe), DESEq2 and EdgeR BioMarker analyses were conducted to search for distinctive zOTUs for each larval gut region, regardless of the fed diet. Only those zOTUs are depicted that were found to be significant for all three analyses. The EF-LDA score for the LEfSe analysis is then shown, along with the taxonomy (at genus level) of the specific zOTU.

To strengthen the above observations, three BioMarker analyses (LEfSe, DESeq2, and EdgeR) were used to look for distinctive zOTUs in the larval gut per region. Only if zOTUs were identified by all three methods, they were deemed distinctive for that region. Finally, the analysis was performed both to look at distinct zOTUs in each region, regardless of diet (Figure 7) and on each diet (Supplementary Tables 6, 7). Interestingly, aside from the *Proteus* sp. (zOTU 21), all distinctive zOTUs in the MMG belong to genera known for a higher acid tolerance, which is in line with their ability to survive selection in this low pH micro-environment. This could also explain why it contains much more distinctive zOTUs, compared to the other three regions that exhibit more neutral to basic pH environments (pH 6–8).

**FIGURE 8:**
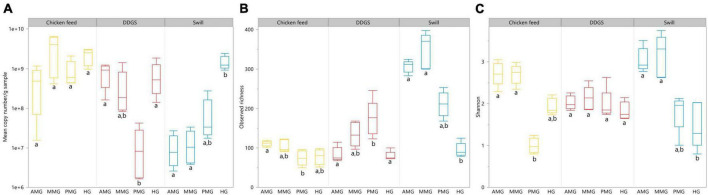
Absolute abundance in 16S rRNA gene copies/g sample **(A)**, observed richness **(B)** and Shannon diversity **(C)** for the black soldier fly larvae gut regions, each for the three different rearing substrates used in this study. Data are presented as boxplots from five replicas. Significant differences between gut regions per substrate are indicated with different letters below the boxplots.

**FIGURE 9:**
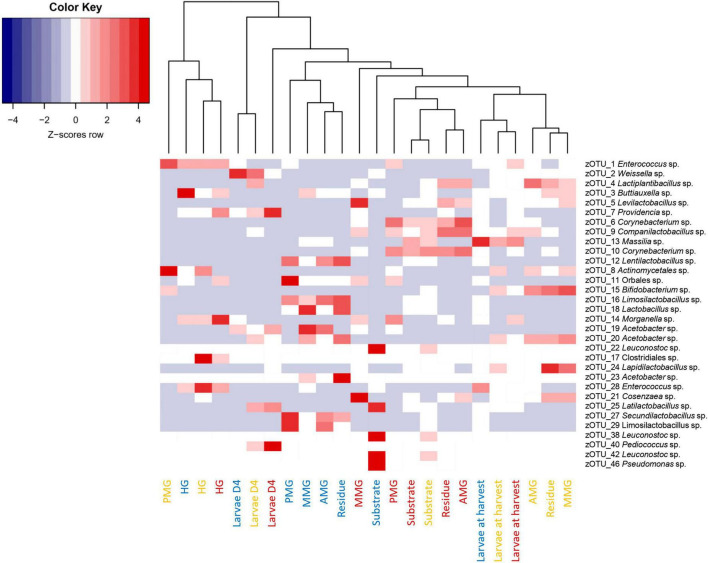
Heatmap representation of the normalized (Z-scores) mean (*n* = 5), except for the chicken feed substrate (*n* = 1), the DDGS substrate (*n* = 4) and the larvae at harvest on DDGS (*n* = 3) relative abundance data for all samples investigated in this study. The heatmap color ranges from blue (negative Z-scores) to red (positive Z-scores). Each row represents a zOTU with an overall relative abundance above 5%, while each column represents a sample. Sample colors refer to the different rearing substrates used (yellow for chicken feed, red for DDGS, blue for swill). Samples are clustered according to a dendrogram, indicating their similarity in bacterial community composition.

Aside from the relative composition of the microbial community in the four gut regions (Figure 5), the total abundance of bacterial DNA (Figure 8A) and diversity indices were determined (Figures 8B, C). Bruno et al. (2019b) reported an increase in bacterial load from the anterior to the posterior region of the midgut, but this trend was only observed for swill diet in this study (Figure 8A). The same is true for the decrease in species diversity (Figures 8B, C). However, for unclear reasons, these trends were far less pronounced on CF and DDGS diet (Figure 8). Nevertheless, a low bacterial variability (Figures 5, 8B, C), but a high total bacterial load (Figure 8A), was detected in the HG samples from all substrates. Additionally, the bacterial biota composition in all HG samples was observed to be very similar, according to NMDS (Figure 6) and heatmap (Figure 9) analysis. A BioMarker analysis among diets for each of the gut regions also points at a decreasing diversity, as fewer distinctive zOTUs are observed when moving along the intestinal tract on each of the diets (Supplementary Table 6). Dominant genera observed in the different HG samples were *Buttiauxella* (zOTU 3), *Enterococcus* (zOTU 1 and 28), *Providencia* (zOTU 7), and *Morganella* (zOTU 14), of which the latter three are named as important members of the “core” microbiota of the BSF (IJdema et al., 2022). The *Providencia* sp. was also identified by the BioMarker analysis to be a distinctive zOTU for the HG region (Figure 7). From these core genera, for example *Buttiauxella* was reported to assist in the hydrolyzation of phytate, a difficult-to-digest component, present in vegetables and especially grains, that inhibits mineral absorption (Shi et al., 2008). The potential role of *Morganella* in nitrogen recycling has already been discussed. For example, zOTU 14 could be further identified (via a BLAST search against GenBank) as *Morganella morganii* and was most abundant in the HG of the larvae at harvest fed on DDGS (13.1% of total counts). This is not surprising as the nitrogenous waste is excreted by the Malpighian tubules at the onset of the HG.

The apparent conserved community in the HG is a feature that is present in other insects as well (Breznak, 2003; Andert et al., 2010). In the cockroach *Periplaneta americana*, the hindgut microbiota presents a highly stable core microbial community with not only a low variety in composition among individuals, but also in response to dietary shifts (Tinker and Ottesen, 2016). These observations seem to indicate a role of the hindgut as a reservoir to harbor or maintain a “core” microbiota, which proliferates depending on the nutrients present in the substrate to help BSF larvae digest remaining recalcitrant macronutrients (e.g., fibers) or recover nutrients from excretion products of metabolism (e.g., uric acid). Evidence of this proliferation is clear when looking at the total abundance of bacterial DNA, which was highest for all diets in the HG region (Figure 8).

As a final side note, in the HG samples obtained from BSF larvae reared on CF, also a large relative abundance of a Clostridiales sp. (zOTU 17, 20%) was observed. As this order contains several endospore-forming species, including potentially pathogenic *Clostridium* spp., this may indicate the ability of bacterial endospores to survive a passage through the midgut regions and all its microbiological barriers to eventually colonize the hindgut. This observation may have food safety implications regarding the further use of BSF larvae as food and feed (Vandeweyer et al., 2021).

## Conclusion

This study generated additional data on the composition and dynamics of the bacterial community in BSF larvae as a whole and along their gut regions, fed on relevant agro-industrial residual streams. Our findings support previous studies mapping these bacterial communities and help to better understand the spatial dynamics of the gut microbiota along the intestinal tract. First, the growth of the larvae on the different diets was analyzed and growth was comparable on DDGS and chicken feed (maximal larval mass of 303 ± 11 mg and 290 ± 11 mg, respectively), but reduced on swill (maximal larval mass of 187 ± 8 mg). A nutritional analysis of the different substrates indicates that the lower protein and carbohydrate content of the swill are likely behind this observation, confirming the, already stated, importance of these nutrients for BSF growth. Zooming in on the microbial community in the whole larvae, two observations can be made. First of all, a shift in the microbial community when larvae are fed on different diets is observed, as stated by several prior studies. However, even when fed on one diet a clear maturation of the microbial community, which entails a diversification as well as a shift of the community, occurs during larval development from day 4 to the end of last instar (day 14 or 17, depending on the diet). Overall, it remains impossible to untangle the impact of these different parameters. More studies including controlled experiments to explore the impact of each single parameter (e.g., nutritional and moisture content of the diet, pH, and larval development) are needed to come to a comprehensive understanding of the dynamics in the bacterial biota. One such experiment could be to rear larvae on a frequently replaced diet to avoid alterations of the diet (e.g., pH increase and moisture content decrease over time) and thus allow a more clear study of how larval aging affects the gut microbiota.

This became even more apparent when exploring the bacterial biota in the different gut regions, which included, for the first time, an analysis of the hindgut region, which has been completely neglected so far as the whole gut or specific midgut districts only have been investigated in previous studies. Overall, most results on abundance and diversity of the bacterial community correspond with those obtained by Bruno et al. (2019b) and indicate selective processes in the BSF midgut that shape the microbial community. A more abundant, but less diverse bacterial community, was observed in the PMG when fed on chicken feed and swill, but not on DDGS. In the hindgut there was an abundant community with low diversity on all diets. This less diverse community was conserved over the three diets and mainly contained *Buttiauxella*, *Enterococcus*, *Providencia*, and *Morganella*, of which the latter three are named as important members of the “core” microbiota of the BSF over different studies (IJdema et al., 2022). We therefore postulate a potential role of the hindgut as a reservoir for insect-associated microbes, warranting further research on that underexplored region of the intestinal tract. Indeed, while the hindgut is mainly associated with osmoregulation and excretion, the conserved presence of specific microorganisms could point at additional functions for example in the processing of recalcitrant fractions of the substrate. For example, bacteria capable of degrading polysaccharides, such as hemicellulose and cellulose, can reside in the insect hindgut (Cook et al., 2007).

Overall, these findings contribute to our understanding of the bacterial biota structure and dynamics along the BSF intestinal tract, which can aid microbiome engineering efforts to enhance larval performance on industrial by-products.

## Data availability statement

The datasets presented in this study can be found in online repositories. The names of the repository/repositories and accession number(s) can be found below: https://www.ncbi.nlm.nih.gov/, PRJNA706185.

## Ethics statement

Ethical approval was not required for the study involving animals in accordance with the local legislation and institutional requirements because this is not required for insects.

## Author contributions

DV, DB, and JD designed the study and wrote the original manuscript. DV, DB, JD, and MB conducted the experimental work and data analysis. FI, SC, and BL assisted in processing and interpretation of sequencing results. MC and GT contributed with manuscript revision, ideas, feedback, and writing throughout manuscript development. All authors contributed to the article and approved the submitted version.

## Supplementary material

Supplementary Figure 1 Non-metric multidimensional scaling (NMDS) plot based on Bray-Curtis dissimilarities derived from the Hellinger transformed relative abundance data of the bacterial communities (stress = 0.1129287) of the black soldier fly larvae at day 4 (D4) during rearing and the larvae at harvest, each for the three different rearing substrates used in this study (*n* = 5), except for the larvae at harvest on DDGS (*n* = 3). The greater the distance between two data points, the more dissimilar the bacterial communities. Different colors represent the different rearing substrates (yellow for chicken feed, red for DDGS, blue for swill), while different symbols represent the different sample types (balls for larvae samples at D4 and circles for larvae samples at harvest).

Supplementary Figure 2 Non-metric multidimensional scaling (NMDS) plot based on Bray-Curtis dissimilarities derived from the Hellinger transformed relative abundance data of the bacterial communities (stress = 0.08969413) of the substrate and the residue after rearing, each for the three different rearing substrates used in this study (*n* = 5), except for the chicken feed substrate (*n* = 1) and the DDGS substrate (*n* = 4). The greater the distance between two data points, the more dissimilar the bacterial communities. Different colors represent the different rearing substrates (yellow for chicken feed, red for DDGS, blue for swill), while different symbols represent the different sample types (triangle for substrate samples and squares for residue samples).

## References

[B1] Andert J. Marten A. Brandl R. Brune A. (2010). Inter- and intraspecific comparison of the bacterial assemblages in the hindgut of humivorous scarab beetle larvae (Pachnoda spp.). *FEMS Microbiol. Ecol.* 74 439–449. 10.1111/J.1574-6941.2010.00950.X 20738398

[B2] Auger L. Deschamps M. H. Vandenberg G. Derome N. (2023). Microbiota is structured by gut regions, life stage, and diet in the Black Soldier Fly (Hermetia illucens). *Front. Microbiol.* 14:1221728. 10.3389/FMICB.2023.1221728 37664118 PMC10469785

[B3] Barragán-Fonseca K. B. Gort G. Dicke M. van Loon J. J. A. (2020). Nutritional plasticity of the black soldier fly (*Hermetia illucens*) in response to artificial diets varying in protein and carbohydrate concentrations. *J. Insects Food Feed* 7 51–61. 10.3920/JIFF2020.0034 29510743

[B4] Beller L. Deboutte W. Falony G. Vieira-Silva S. Tito R. Y. Valles-Colomer M. (2021). Successional stages in infant gut microbiota maturation. *mBio* 12:e0185721. 10.1128/mBio.01857-21 34903050 PMC8686833

[B5] Beniers J. J. A. Graham R. I. (2019). Effect of protein and carbohydrate feed concentrations on the growth and composition of black soldier fly (Hermetia illucens) larvae. *J. Insects Food Feed* 5 193–199. 10.3920/JIFF2018.0001 29510743

[B6] Bonelli M. Bruno D. Brilli M. Gianfranceschi N. Tian L. Tettamanti G. (2020). Black soldier fly larvae adapt to different food substrates through morphological and functional responses of the midgut. *Int. J. Mol. Sci.* 21:4955. 10.3390/IJMS21144955 32668813 PMC7404193

[B7] Bonelli M. Bruno D. Caccia S. Sgambetterra G. Cappellozza S. Jucker C. (2019). Structural and functional characterization of Hermetia illucens larval midgut. *Front. Physiol.* 10:204. 10.3389/fphys.2019.00204 30906266 PMC6418021

[B8] Borremans A. Crauwels S. Vandeweyer D. Smets R. Verreth C. Van Der Borght M. (2019). Comparison of six commercial meat starter cultures for the fermentation of yellow mealworm (Tenebrio molitor) paste. *Microorganisms* 7:540. 10.3390/microorganisms7110540 31717367 PMC6920846

[B9] Bossaert S. Winne V. Van Opstaele F. Buyse J. Verreth C. Herrera-Malaver B. (2021). Description of the temporal dynamics in microbial community composition and beer chemistry in sour beer production via barrel ageing of finished beers. *Int. J. Food Microbiol.* 339:109030. 10.1016/j.ijfoodmicro.2020.109030 33387813

[B10] Breznak J. A. (2003). Intestinal microbiota of termites and other xylophagous insects. *Annu. Rev. Microbiol.* 36 323–343. 10.1146/ANNUREV.MI.36.100182.001543 6756291

[B11] Broeckx L. Frooninckx L. Slegers L. Berrens S. Noyens I. Goossens S. (2021). Growth of black soldier fly larvae reared on organic side-streams. *Sustainability* 13:12953. 10.3390/SU132312953

[B12] Bruno D. Bonelli M. Cadamuro A. G. Reguzzoni M. Grimaldi A. Casartelli M. (2019a). The digestive system of the adult Hermetia illucens (Diptera: Stratiomyidae): Morphological features and functional properties. *Cell Tissue Res.* 378 221–238. 10.1007/S00441-019-03025-7 31053891

[B13] Bruno D. Bonelli M. De Filippis F. Di Lelio I. Tettamanti G. Casartelli M. (2019b). The intestinal microbiota of hermetia illucens larvae is affected by diet and shows a diverse composition in the different midgut regions. *Appl. Environ. Microbiol.* 85:e0186418. 10.1128/AEM.01864-18 30504212 PMC6328772

[B14] Callegari M. Jucker C. Fusi M. Leonardi M. G. Daffonchio D. Borin S. (2020). Hydrolytic profile of the culturable gut bacterial community associated with Hermetia illucens. *Front. Microbiol.* 11:1965. 10.3389/FMICB.2020.01965 32903451 PMC7434986

[B15] Caporaso J. G. Lauber C. L. Walters W. A. Berg-Lyons D. Lozupone C. A. Turnbaugh P. J. (2011). Global patterns of 16S rRNA diversity at a depth of millions of sequences per sample. *Proc. Natl. Acad. Sci. U.S.A.* 108 4516–4522. 10.1073/pnas.1000080107 20534432 PMC3063599

[B16] Cappellozza S. Leonardi M. G. Savoldelli S. Carminati D. Rizzolo A. Cortellino G. (2019). A first attempt to produce proteins from insects by means of a circular economy. *Animals* 9:278. 10.3390/ani9050278 31137732 PMC6562786

[B17] Ceccotti C. Bruno D. Tettamanti G. Branduardi P. Bertacchi S. Labra M. (2022). New value from food and industrial wastes – Bioaccumulation of omega-3 fatty acids from an oleaginous microbial biomass paired with a brewery by-product using black soldier fly (Hermetia illucens) larvae. *Waste Manag.* 143 95–104. 10.1016/J.WASMAN.2022.02.029 35240451

[B18] Cook D. M. Henriksen E. D. C. Upchurch R. Doran Peterson J. B. (2007). Isolation of polymer-degrading bacteria and characterization of the hindgut bacterial community from the detritus-feeding larvae of Tipula abdominalis (Diptera: Tipulidae). *Appl. Environ. Microbiol.* 73 5683–5686. 10.1128/AEM.00213-07 17630316 PMC2042085

[B19] Davis N. M. Proctor D. M. Holmes S. P. Relman D. A. Callahan B. J. (2018). Simple statistical identification and removal of contaminant sequences in marker-gene and metagenomics data. *Microbiome* 6:226. 10.1186/s40168-018-0605-2 30558668 PMC6298009

[B20] De Smet J. Vandeweyer D. Van Moll L. Lachi D. Van Campenhout L. (2021). Dynamics of *Salmonella* inoculated during rearing of black soldier fly larvae (Hermetia illucens). *Food Res. Int.* 149:110692. 10.1016/J.FOODRES.2021.110692 34600687 PMC8505792

[B21] De Smet J. Wynants E. Cos P. Van Campenhout L. (2018). Microbial community dynamics during rearing of black soldier fly larvae (Hermetia illucens) and impact on exploitation potential. *Appl. Environ. Microbiol.* 84 e02722–17. 10.1128/AEM.02722-17 29475866 PMC5930328

[B22] Dillon R. J. Dillon V. M. (2004). The gut bacteria of insects: Nonpathogenic interactions. *Annu. Rev. Entomol.* 49 71–92. 10.1146/annurev.ento.49.061802.123416 14651457

[B23] Douglas A. E. (2015). Multiorganismal insects: Diversity and function of resident microorganisms. *Annu. Rev. Entomol.* 60 17–34. 10.1146/ANNUREV-ENTO-010814-020822 25341109 PMC4465791

[B24] Edgar R. C. (2013). UPARSE: Highly accurate OTU sequences from microbial amplicon reads. *Nat. Methods* 10 996–998. 10.1038/nmeth.2604 23955772

[B25] Edgar R. C. (2016). UNOISE2: Improved error-correction for Illumina 16S and ITS amplicon sequencing. *bioRxiv [Preprint]* 10.1101/081257

[B26] Edgar R. C. Flyvbjerg H. (2015). Error filtering, pair assembly and error correction for next-generation sequencing reads. *Bioinformatics* 31 3476–3482. 10.1093/bioinformatics/btv401 26139637

[B27] Eggink K. M. Donoso I. G. Dalsgaard J. (2023). Optimal dietary protein to carbohydrate ratio for black soldier fly (Hermetia illucens) larvae. *J. Insects Food Feed* 9 789–798. 10.3920/JIFF2022.0102 29510743

[B28] Engel P. Moran N. A. (2013). The gut microbiota of insects - diversity in structure and function. *FEMS Microbiol. Rev.* 37 699–735. 10.1111/1574-6976.12025 23692388

[B29] Everaert N. Van Cruchten S. Weström B. Bailey M. Van Ginneken C. Thymann T. (2017). A review on early gut maturation and colonization in pigs, including biological and dietary factors affecting gut homeostasis. *Anim. Feed Sci. Technol.* 233 89–103. 10.1016/J.ANIFEEDSCI.2017.06.011

[B30] Gloder G. Bourne M. E. Verreth C. Wilberts L. Bossaert S. Crauwels S. (2021). Parasitism by endoparasitoid wasps alters the internal but not the external microbiome in host caterpillars. *Anim. Microbiome* 3:73. 10.1186/S42523-021-00135-Y 34654483 PMC8520287

[B31] Gorrens E. De Smet J. Vandeweyer D. Bossaert S. Crauwels S. Lievens B. (2022). The bacterial communities of black soldier fly larvae (Hermetia illucens) during consecutive, industrial rearing cycles. *J. Insects Food Feed* 8 1061–1076. 10.3920/JIFF2021.0150 29510743

[B32] Gorrens E. Van Looveren N. Van Moll L. Vandeweyer D. Lachi D. De Smet J. (2021). *Staphylococcus aureus* in substrates for black soldier fly larvae (Hermetia illucens) and its dynamics during rearing. *Microbiol. Spectr.* 9:e0218321. 10.1128/spectrum.02183-21 34937197 PMC8694120

[B33] Green T. R. Popa R. (2012). Enhanced ammonia content in compost leachate processed by black soldier fly larvae. *Appl. Biochem. Biotechnol.* 166 1381–1387. 10.1007/s12010-011-9530-6 22238016

[B34] Guo X. Liu N. Li X. Ding Y. Shang F. Gao Y. (2015). Red soils harbor diverse culturable actinomycetes that are promising sources of novel secondary metabolites. *Appl. Environ. Microbiol.* 81:3086. 10.1128/AEM.03859-14 25724963 PMC4393440

[B35] Heussler C. D. Klammsteiner T. Stonig K. T. Insam H. Schlick-Steiner B. C. Steiner F. M. (2022). Decrypting the microbiota on the black soldier fly’s (Hermetia illucens L., Diptera: Stratiomyidae) egg surface and their origin during development. *bioRxiv [Preprint]* 10.1101/2022.12.22.520758

[B36] Horie Y. Watanabe K. (1983). Effect of various kinds of dietary protein and supplementation with limiting amino acids on growth, haemolymph components and uric acid excretion in the silkworm, Bombyx mori. *J. Insect Physiol.* 29 187–199. 10.1016/0022-1910(83)90143-9

[B37] Horwitz W. (ed.) (2000). *Official methods of analysis of AOAC International.* Revision 1, 17th Edn. Gaithersburg MD: AOAC International.

[B38] IJdema F. de Smet J. Crauwels S. Lievens B. van Campenhout L. (2022). Meta-analysis of larvae of the black soldier fly (Hermetia illucens) microbiota based on 16S rRNA gene amplicon sequencing. *FEMS Microbiol. Ecol.* 98:fiac094. 10.1093/FEMSEC/FIAC094 35977400 PMC9453823

[B39] Iram A. Cekmecelioglu D. Demirci A. (2020). Distillers’ dried grains with solubles (DDGS) and its potential as fermentation feedstock. *Appl. Microbiol. Biotechnol.* 104 6115–6128. 10.1007/S00253-020-10682-0/TABLES/4 32440706

[B40] Jeon H. Park S. Choi J. Jeong G. Lee S.-B. Choi Y. (2011). The intestinal bacterial community in the food waste-reducing larvae of Hermetia illucens. *Curr. Microbiol.* 62 1390–1399. 10.1007/s00284-011-9874-8 21267722

[B41] Jiang C. L. Jin W. Z. Tao X. H. Zhang Q. Zhu J. Feng S. Y. (2019). Black soldier fly larvae (Hermetia illucens) strengthen the metabolic function of food waste biodegradation by gut microbiome. *Microb. Biotechnol.* 12 528–543. 10.1111/1751-7915.13393 30884189 PMC6465238

[B42] Klammsteiner T. Walter A. Bogataj T. Heussler C. D. Stres B. Steiner F. M. (2020). The core gut microbiome of black soldier fly (Hermetia illucens) larvae raised on low-bioburden diets. *Front. Microbiol.* 11:993. 10.3389/FMICB.2020.00993 32508795 PMC7253588

[B43] Klammsteiner T. Walter A. Bogataj T. Heussler C. D. Stres B. Steiner F. M. (2021). Impact of processed food (Canteen and Oil Wastes) on the development of black soldier fly (Hermetia illucens) larvae and their gut microbiome functions. *Front. Microbiol.* 12:619112. 10.3389/FMICB.2021.619112 33552039 PMC7858275

[B44] Kozich J. J. Westcott S. L. Baxter N. T. Highlander S. K. Schloss P. D. (2013). Development of a dual-index sequencing strategy and curation pipeline for analyzing amplicon sequence data on the MiSeq Illumina sequencing platform. *Appl. Environ. Microbiol.* 79 5112–5120. 10.1128/AEM.01043-13 23793624 PMC3753973

[B45] Kwong W. K. Moran N. A. (2013). Cultivation and characterization of the gut symbionts of honey bees and bumble bees: Description of Snodgrassella alvi gen. nov., sp. nov., a member of the family Neisseriaceae of the betaproteobacteria, and Gilliamella apicola gen. nov., sp. nov., a member of Orbaceae fam. nov., Orbales ord. nov., a sister taxon to the order “*Enterobacteriales*” of the Gammaproteobacteria. *Int. J. Syst. Evol. Microbiol.* 63 2008–2018. 10.1099/IJS.0.044875-0 23041637

[B46] Laganaro M. Bahrndorff S. Eriksen N. T. (2021). Growth and metabolic performance of black soldier fly larvae grown on low and high-quality substrates. *Waste Manag.* 121 198–205. 10.1016/J.WASMAN.2020.12.009 33360818

[B47] Latimer G. W. (ed.) (2016). *Official methods of analysis of AOAC international*, 20th Edn. Rockville, MD: AOAC International.

[B48] Lievens S. Poma G. Frooninckx L. Van der Donck T. Seo J. W. De Smet J. (2022). Mutual Influence between Polyvinyl Chloride (Micro)Plastics and black soldier fly larvae (Hermetia illucens L.). *Sustainability* 14:12109. 10.3390/SU141912109/S1 37577731

[B49] Martinson V. G. Carpinteyro-Ponce J. Moran N. A. Markow T. A. (2017). A distinctive and host-restricted gut microbiota in populations of a cactophilic Drosophila species. *Appl. Environ. Microbiol.* 83 e01551–17. 10.1128/AEM.01551-17 28939605 PMC5691420

[B50] McMurdie P. J. Holmes S. (2013). phyloseq: An R package for reproducible interactive analysis and graphics of microbiome census data. *PLoS One* 8:e61217. 10.1371/JOURNAL.PONE.0061217 23630581 PMC3632530

[B51] Menconi A. Kallapura G. Latorre J. D. Morgan M. J. Pumford N. R. Hargis B. M. (2014). Identification and characterization of lactic acid bacteria in a commercial probiotic culture. *Biosci. Microb. Food Health* 33:25. 10.12938/BMFH.33.25 24936379 PMC4034328

[B52] Meneguz M. Gasco L. Tomberlin J. K. (2018). Impact of pH and feeding system on black soldier fly (Hermetia illucens, L; Diptera: Stratiomyidae) larval development. *PLoS One* 13:e0202591. 10.1371/JOURNAL.PONE.0202591 30148867 PMC6110483

[B53] Mubarak Z. Soraya C. (2018). The acid tolerance response and pH adaptation of Enterococcus faecalis in extract of lime Citrus aurantiifolia from Aceh Indonesia. *F1000Research* 7:287. 10.12688/F1000RESEARCH.13990.2 29721312 PMC5897787

[B54] Nguyen T. T. X. Tomberlin J. K. Vanlaerhoven S. (2013). Influence of resources on Hermetia illucens (Diptera: Stratiomyidae) larval development. *J. Med. Entomol.* 50 898–906. 10.1603/ME12260 23926790

[B55] Oksanen J. Blanchet F. G. Friendly M. Kindt R. Legendre P. McGlinn D. (2022). *vegan: Community Ecology Package. R package version 2.5-7. 2020.* Available online at: https://www.researchgate.net/publication/346579465_vegan_community_ecology_package_version_25-7_November_2020

[B56] Padmanabha M. Kobelski A. Hempel A. J. Streif S. (2020). A comprehensive dynamic growth and development model of Hermetia illucens larvae. *PLoS One* 15:e0239084. 10.1371/JOURNAL.PONE.0239084 32946462 PMC7500678

[B57] R Development Core Team (2013). *R: A language and environment for statistical computing.* Vienna: R Foundation for Statistical Computing.

[B58] Ren X. Cao S. Akami M. Mansour A. Yang Y. Jiang N. (2022). Gut symbiotic bacteria are involved in nitrogen recycling in the tephritid fruit fly Bactrocera dorsalis. *BMC Biol.* 20:201. 10.1186/S12915-022-01399-9 36104720 PMC9476588

[B59] Scala A. Cammack J. A. Salvia R. Scieuzo C. Franco A. Bufo S. A. (2020). Rearing substrate impacts growth and macronutrient composition of Hermetia illucens (L.) (Diptera: Stratiomyidae) larvae produced at an industrial scale. *Sci. Rep.* 10 1–8. 10.1038/s41598-020-76571-8 33173088 PMC7655861

[B60] Shi P. Huang H. Wang Y. Luo H. Wu B. Meng K. (2008). A novel phytase gene appA from Buttiauxella sp. GC21 isolated from grass carp intestine. *Aquaculture* 275 70–75. 10.1016/J.AQUACULTURE.2008.01.021

[B61] Shumo M. Khamis F. M. Ombura F. L. Tanga C. M. Fiaboe K. K. M. Subramanian S. (2021). A molecular survey of bacterial species in the guts of black soldier fly larvae (Hermetia illucens) reared on two urban organic waste streams in Kenya. *Front. Microbiol.* 12:687103. 10.3389/FMICB.2021.687103 34630342 PMC8493336

[B62] Starke R. Pylro V. S. Morais D. K. (2021). 16S rRNA gene copy number normalization does not provide more reliable conclusions in metataxonomic surveys. *Microb. Ecol.* 81 535–539. 10.1007/S00248-020-01586-7 32862246 PMC7835310

[B63] Tegtmeier D. Hurka S. Klüber P. Brinkrolf K. Heise P. Vilcinskas A. (2021a). Cottonseed press cake as a potential diet for industrially farmed black soldier fly larvae triggers adaptations of their bacterial and fungal gut microbiota. *Front. Microbiol.* 12:634503. 10.3389/FMICB.2021.634503 33854488 PMC8039154

[B64] Tegtmeier D. Hurka S. Mihajlovic S. Bodenschatz M. Schlimbach S. Vilcinskas A. (2021b). Culture-independent and culture-dependent characterization of the black soldier fly gut microbiome reveals a large proportion of culturable bacteria with potential for industrial applications. *Microorganisms* 9:1642. 10.3390/microorganisms9081642 34442721 PMC8398798

[B65] Tettamanti G. Van Campenhout L. Casartelli M. (2022). A hungry need for knowledge on the black soldier fly digestive system. *J. Insects Food Feed* 8 217–222. 10.3920/JIFF2022.X002 29510743

[B66] Tinker K. A. Ottesen E. A. (2016). The core gut microbiome of the American cockroach, Periplaneta americana, is stable and resilient to dietary shifts. *Appl. Environ. Microbiol.* 82 6603–6610. 10.1128/AEM.01837-16 27590811 PMC5086554

[B67] Tomberlin J. K. van Huis A. (2020). Black soldier fly from pest to “crown jewel” of the insects as feed industry: An historical perspective. *J. Insects Food Feed* 6 1–4. 10.3920/JIFF2020.0003 29510743

[B68] Vandeweyer D. De Smet J. Van Looveren N. Van Campenhout L. (2021). Biological contaminants in insects as food and feed. *J. Insects Food Feed* 7 807–822. 10.3920/JIFF2020.0060 29510743

[B69] Varelas V. (2019). Food wastes as a potential new source for edible insect mass production for food and feed: A review. *Fermentation* 5:81. 10.3390/FERMENTATION5030081

[B70] Warnes M. G. R. Bolker B. Bonebakker L. Gentleman R. Huber W. (2016). *Package ‘gplots.’ Various R programming tools for plotting data.*

[B71] Woods M. J. Hoffman L. C. Pieterse E. (2019). Artificial diets for neonatal black soldier fly (Hermetia illucens) larvae. *J. Insects Food Feed* 5 99–105. 10.3920/JIFF2018.0028 29510743

[B72] Wynants E. Frooninckx L. Crauwels S. Verreth C. De Smet J. Sandrock C. (2019). Assessing the microbiota of black soldier fly larvae (Hermetia illucens) reared on organic waste streams on four different locations at laboratory and large scale. *Microb. Ecol.* 77 913–930. 10.1007/S00248-018-1286-X 30430196

[B73] Yakti W. Müller M. Klost M. Mewis I. Dannehl D. Ulrichs C. (2023). Physical properties of substrates as a driver for Hermetia illucens (L.) (Diptera: Stratiomyidae) larvae growth. *Insects* 14:266. 10.3390/INSECTS14030266 36975951 PMC10054678

[B74] Yu Y. Zhang J. Zhu F. Fan M. Zheng J. Cai M. (2023). Enhanced protein degradation by black soldier fly larvae (Hermetia illucens L.) and its gut microbes. *Front. Microbiol.* 13:1095025. 10.3389/FMICB.2022.1095025 36704554 PMC9871565

[B75] Zhang X. Zhang J. Jiang L. Yu X. Zhu H. Zhang J. (2021). Black soldier fly (Hermetia illucens) larvae significantly change the microbial community in chicken manure. *Curr. Microbiol.* 78 303–315. 10.1007/S00284-020-02276-W 33141316

[B76] Zheng H. Perreau J. Elijah Powell J. Han B. Zhang Z. Kwong W. K. (2019). Division of labor in honey bee gut microbiota for plant polysaccharide digestion. *Proc. Natl. Acad. Sci. U.S.A.* 116 25909–25916. 10.1073/PNAS.1916224116/-/DCSUPPLEMENTAL 31776248 PMC6926048

[B77] Zheng L. Crippen T. L. Singh B. Tarone A. M. Dowd S. Yu Z. (2013). A survey of bacterial diversity from successive life stages of black soldier fly (Diptera: Stratiomyidae) by using 16S rDNA pyrosequencing. *J. Med. Entomol.* 50 647–658. 10.1603/ME12199 23802462

[B78] Zheng L. Hou Y. Li W. Yang S. Li Q. Yu Z. (2012). Biodiesel production from rice straw and restaurant waste employing black soldier fly assisted by microbes. *Energy* 47 225–229. 10.1016/J.ENERGY.2012.09.006

[B79] Zhineng Y. Ying M. Bingjie T. Rouxian Z. Qiang Z. (2021). Intestinal microbiota and functional characteristics of black soldier fly larvae (Hermetia illucens). *Ann. Microbiol.* 71:13. 10.1186/S13213-021-01626-8

