# Supplementary figures and images for: Bacterial biota composition in gut regions of black soldier fly larvae reared on industrial residual streams: revealing community dynamics along its intestinal tract
Source: Front Microbiol. 2023 Dec 1;14:1276187. doi: 10.3389/fmicb.2023.1276187 (PMC10722301; doi:10.3389/fmicb.2023.1276187)

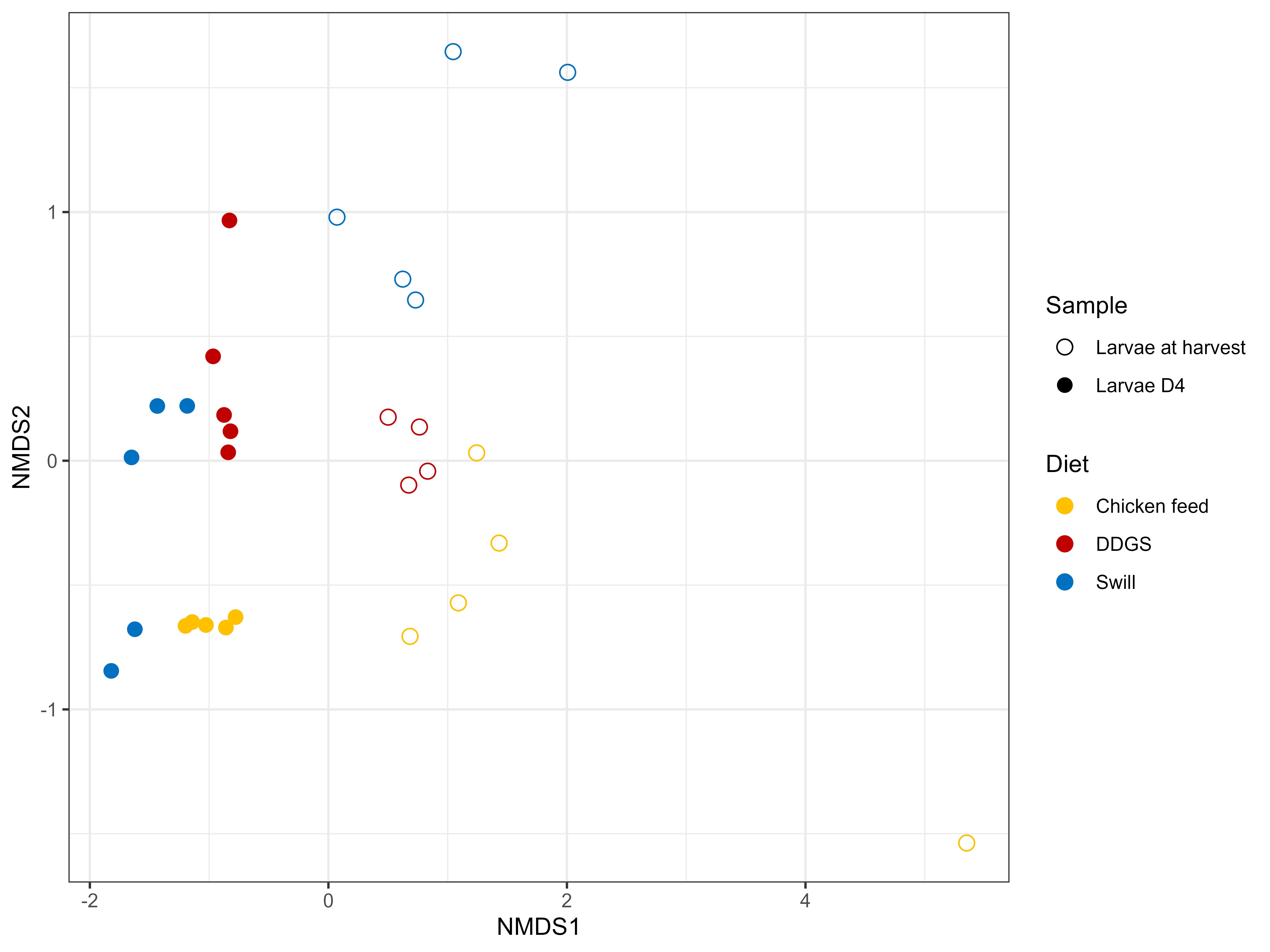

Supplement: Supplementary Figure 1 — Non-metric multidimensional scaling (NMDS) plot based on Bray-Curtis dissimilarities derived from the Hellinger transformed relative abundance data of the bacterial communities (stress = 0.1129287) of the black soldier fly larvae at day 4 (D4) during rearing and the larvae at harvest, each for the three different rearing substrates used in this study (n = 5), except for the larvae at harvest on DDGS (n = 3). The greater the distance between two data points, the more dissimilar the bacterial communities. Different colors represent the different rearing substrates (yellow for chicken feed, red for DDGS, blue for swill), while different symbols represent the different sample types (balls for larvae samples at D4 and circles for larvae samples at harvest). [file Image_1.JPEG]

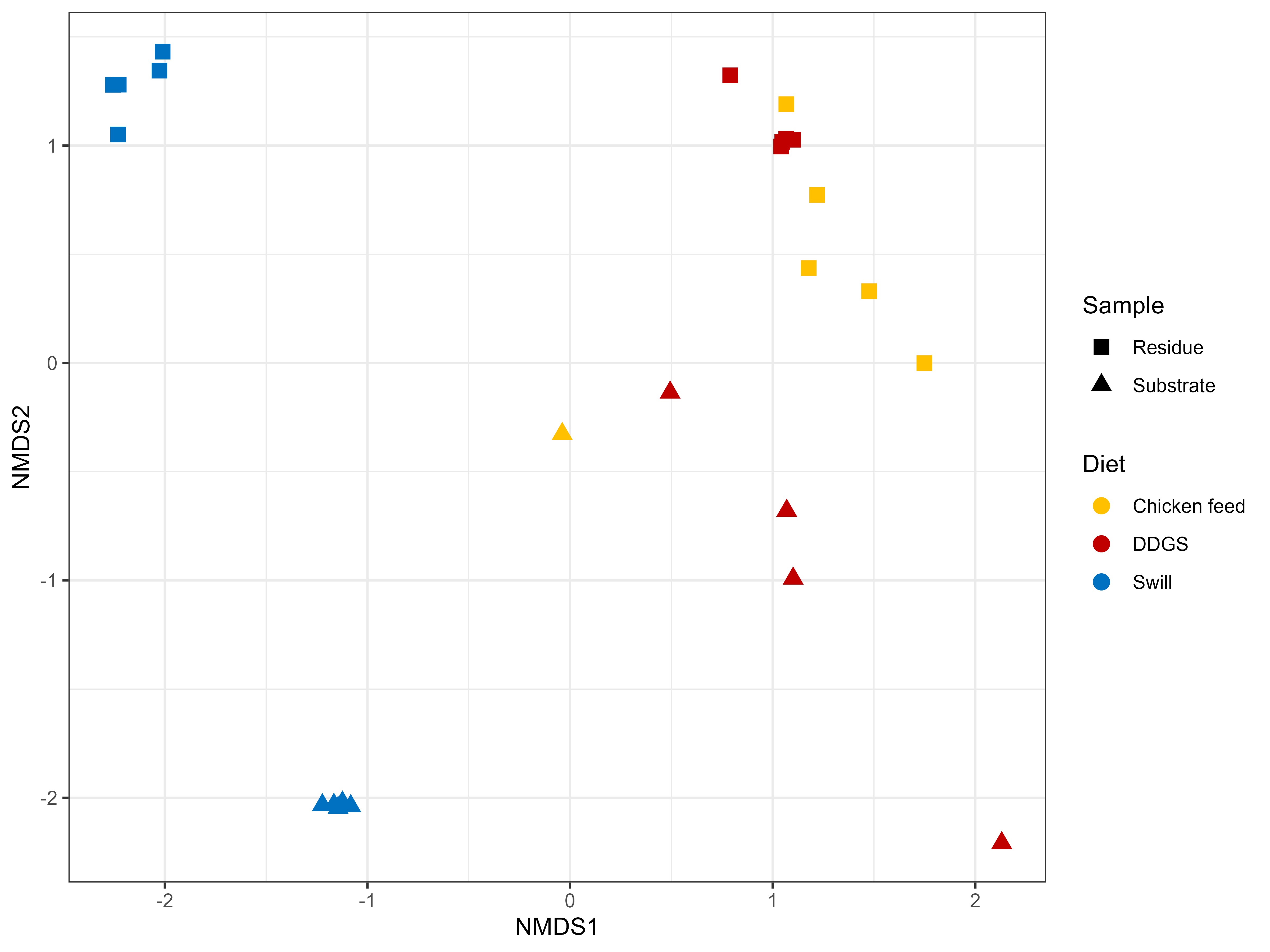

Supplement: Supplementary Figure 2 — Non-metric multidimensional scaling (NMDS) plot based on Bray-Curtis dissimilarities derived from the Hellinger transformed relative abundance data of the bacterial communities (stress = 0.08969413) of the substrate and the residue after rearing, each for the three different rearing substrates used in this study (n = 5), except for the chicken feed substrate (n = 1) and the DDGS substrate (n = 4). The greater the distance between two data points, the more dissimilar the bacterial communities. Different colors represent the different rearing substrates (yellow for chicken feed, red for DDGS, blue for swill), while different symbols represent the different sample types (triangle for substrate samples and squares for residue samples). [file Image_2.jpeg]
